# Comparative endoscopic techniques of medial rectus muscle retraction for approaching intraconal tumors: Our experience with five cases

**DOI:** 10.3389/fsurg.2022.923712

**Published:** 2022-07-15

**Authors:** Linli Chen, Xiaorong Yan, Yunshan Fu, Tingting Wang, Zhiyun Zhan, Shengnan Ye, Changzhen Jiang, Guohao Chen

**Affiliations:** ^1^ Department of Otorhinolaryngology, Fujian Institute of Otorhinolaryngology, The First Affiliated Hospital, Fujian Medical University, Fuzhou, China; ^2^ Department of Neurosurgery, The First Affiliated Hospital, Fujian Medical University, Fuzhou, China; ^3^ Department of Otorhinolaryngology, Minnan Hospital Affiliated to Fujian Medical University, Quanzhou, China; ^4^ Department of Ophthalmology, The First Affiliated Hospital, Fujian Medical University, Fuzhou, China

**Keywords:** intraconal tumors, endoscopic orbital surgery, medial rectus muscle retraction, eyelid speculum, tumors lateral to the optic nerve

## Abstract

**Objective:**

To examine the role of transnasal endoscopic approaches in the management of intraconal tumors and demonstrate the use of an eyelid speculum in comparison with different techniques of medial rectus muscle (MRM) retraction.

**Methods:**

Retrospective data of five patients with intraconal tumors operated on and followed up by the senior authors between December 2019 and April 2022 was collected. Presenting symptoms, technical details, imaging and histology findings, outcomes, and complications were evaluated.

**Results:**

Four primary and one recurrent tumor were identified. The mean patient age was 50 (range, 29–64) years. One tumor was located lateral to the optic nerve, one central and three medial. A complete surgical resection was obtained in four primary cases and a partial resection was achieved in the recurrent case. The MRM was retracted using three different techniques: (1) an infant eyelid speculum creating an operative window between the medial and inferior rectus muscle, (2) external MRM disinsertion transconjunctivally, (3) a four-handed technique performed transseptally by two surgeons. Transient postoperative ophthalmoplegia was recorded in four cases and transient ptosis in one. Three patients completely recovered in 2–3 months while one undergoing MRM disinsertion ended up in restricted strabismus at 15-month follow-up. No other long-term complications have been noted in all five patients with a mean follow-up of 22 (range, 14–32) months. No patients with primary tumors have required additional surgery for tumor recurrence.

**Conclusion:**

The indication of endoscopic intraconal surgery may expand to lesions lateral to the optic nerve when the nerve is not in its natural position. The well-known advantages of the endoscopic techniques, namely the lack of external scars, better visualization, less bleeding, and fewer complications, were confirmed. An eye speculum provides a better surgical corridor and eases the pressure exerted on the MRM, which has a promising application prospect.

## Introduction

Surgical management of intraconal pathology represents the next frontier in endoscopic endonasal surgery. A broad range of tumors can occur in the intraconal space and pose both a diagnostic and surgical challenge because of the narrow, complex anatomy. This region must be approached with great care because of the close proximity of several important anatomical structures including the carotid arteries, ophthalmic artery, optic nerve, oculomotor nerve, and cavernous sinus (1, 2).

Intraconal tumors have been classically approached through external approaches, such as frontotemporal craniotomy with orbitozygomatic osteotomy, transcutaneous or transconjunctival orbitotomy, and lateral orbitotomy approaches (3). The introduction of endoscopic surgery has revolutionized the management of orbital tumors located in the medial and inferior orbit with better surgical exposure and excellent outcomes. The lateral orbit, however, has traditionally been considered unsafe for the endonasal approach.

The medial rectus muscle (MRM) forms the medial boundary to the intraconal space. Techniques of MRM retraction has been continually advancing, from MRM disinsertion to transient medialization of MRM. A safe and effective method of retraction tends to well expose the tumor, keep the integrity of muscles, and minimize postoperative complications. This study presents a retrospective review of endoscopic approaches to intraconal tumors and describes the use of an eyelid speculum with comparison to different techniques of MRM retraction.

## Materials and methods

A retrospective analysis was conducted, entering five patients with intraconal tumors (four primary and one recurrent) operated on and followed up by the senior authors between December 2019 and April 2022. All the patients went through an endoscopic approach. The MRM was retracted using three different techniques: (1) an infant eyelid speculum creating an operative window between the medial and inferior rectus muscle (IRM), (2) external MRM disinsertion via a transconjunctival incision, and (3) a four-handed method performed transseptally by two surgeons.

Presenting symptoms, technical details, imaging and histology findings, and outcomes and complications were evaluated.

## Results

**Figure 1:**
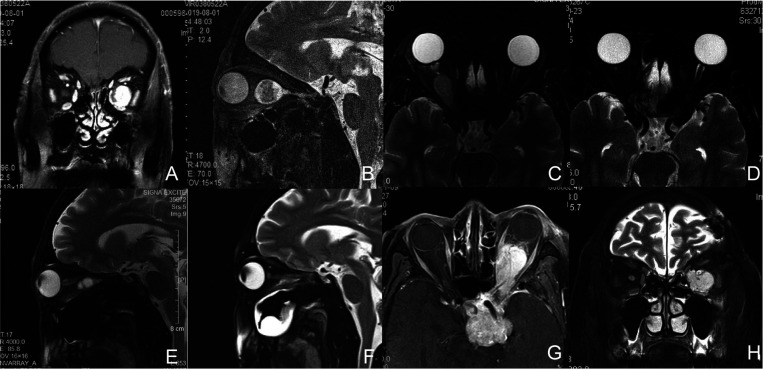
Magnetic resonance (MR) images of Patient1, 2, 3, and 5. (**A,B**) Preoperative coronal (**A**) and sagittal (**B**) T2-weighted images of Patient 1 with a left intraconal schwannoma medial to the optic nerve. (**C**,**D**) Axial T2-weighted images of Patient 2 with a right intraconal meningioma involving the intracanalicular segment of the optic nerve preoperatively (**C**) and 2 months postoperatively (**D**). (**E**,**F**) Sagittal T2-weighted images of Patient 3 with a right intraconal schwannoma preoperatively (**E**) and 2 months postoperatively (**F**). (**G**,**H**) Axial T1-weighted (**G**) and coronal T2-weighted (**H**) images of Patient 5 with a recurrent pituitary tumor involving the sellar region, suprasellar region, cavernous sinus, intraconal space, infratemporal fossa, and pterygopalatine fossa.

**Figure 2:**
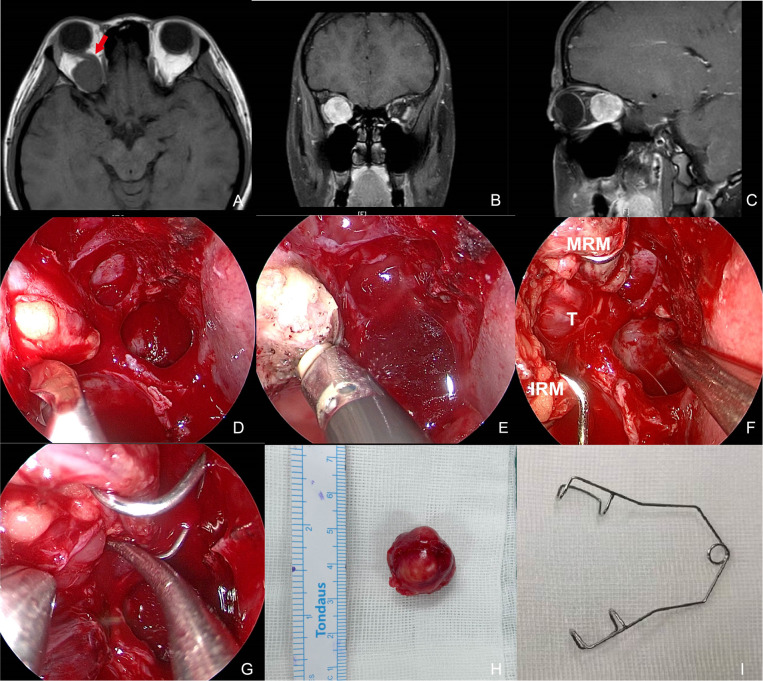
Preoperative MR images and intraoperative pictures of Patient 4 with a schwannoma lateral to the optic nerve. (**A**–**C**) Preoperative MR images in axial (**A**), coronal (**B**), and sagittal (**C**) view. The red arrow indicates the optic nerve is located superomedially. (**D**) Periorbita resected by a sickle knife. (**E**) Extraconal fat was removed using low-temperature plasma. (**F**) Intraoperative exposure of the tumor by an eye speculum placed between the MRM and IRM. (**G**) Blunt dissection of the tumor. (**H**) Completely-resected tumor specimen. (**I**) Photograph of an eye speculum. MRM, medial rectus muscle; IRM, inferior rectus muscle; T, tumor.

A retrospective chart review of five patients with intraconal tumors undergoing transnasal endoscopic surgery was conducted (Table 1). There were two males and three females, with a mean age of 50 (range, 29–64) years. Two patients were referred from neurology and three from ophthalmology. Four primary tumors and one recurrent pituitary tumor were identified (Figures 1, 2). The mean tumor size was 2.1 cm in maximum dimension (range, 2.0–2.5 cm). One tumor was located lateral to the optic nerve, one central, and two medial. There was one left-sided tumor and three right-sided. Total resection was obtained in all primary tumors, while partial resection was achieved in the recurrent case.

**Table 1:** Summary of patient findings.

Pt	Sex, age at op (years)	Pre op findings	Hystology	Side	Location	Size (cm)	Approach	MRM retraction technique	Procedure	Pre op VA	Po op VA	Po op complications	FU (m)
1	M,64	VI, prop	Schwan	L	Medial	2	Bi	4-handed way	TR	0.4 OS	0.6 OS	Transient ophthalmoplegia	32
2	F,42	VI, prop	Meni	R	Central	2	Uni	Disinsertion	TR	NLP OS	NLP OS	Transient ptosis, ophthalmoplegia	25
3	M,51	VI	Schwan	R	Medial	2	Uni	Disinsertion	TR	0.15 OS	0.5 OS	Restricted strabismus	23
4	F,29	VI, ophthalmalgia	Schwan	R	Lateral	2.5	Uni	Speculum	TR	0.6 OD	1.0 OD	Transient ophthalmoplegia	14
5	F,64	Ophthalmalgia, prop	Pituitary endocrine tumor	L	Medial	-	Uni	Speculum	SR	NLP OS	NLP OS	Persistence of disease (treated with Gamma Knife)	16

*Pt, patient; M, male; F, female; Pre op, preoperative; VI, visual impairment; prop, proptosis; Schwan, Schwannoma; Haem, haemangioma; Meni, meningioma; Bi, binarial; Uni, uninarial; MRM, medial rectus muscle; TR, total resection; SR, subtotal resection; VA, visual acuity; Po op, postoperative; NLP, no light perception; FU, follow up.*

Patient 1 complained of decreased vision and a slight protrusion of the left eye. Physical examination revealed left eye proptosis and normal motility. His visual acuity was 0.6OD and 0.4OS. MRI revealed a retroocular mass closely related to the optic nerve in his left orbit. The lesion showed an obvious uneven contrast enhancement (Figures 1A,B). Intraoperatively, the lesion was identified medial to the optic nerve and completely resected. The MRM was retracted using a four-handed technique. Histopathology was consistent with schwannoma. Visual acuity increased to 0.6OD and 0.6OS following surgery. The patient experienced a transient restriction of adduction and suppression in his left eye, and then completely recovered in 3 months.

Patient 2 presented with a 6-month history of progressively decreased vision in her right eye. Her visual acuity was no light perception OD and 0.6 OS. Loss of pupillary light reflex was detected in her right eye. Extraocular muscle function was normal, and there was 1.8 mm of axial right proptosis. MRI demonstrated a lesion in the central orbit with homogeneous enhancement (Figure 1C). MRM was detached externally for access to the tumor. Intraoperative findings showed the lesion involved the intracanalicular segment of the optic nerve. The optic nerve was cut off to allow for complete resection of the tumor. The diagnosis was consistent with meningioma. Visual acuity remained unchanged after surgery. There was transient right ptosis and restriction of adduction and suppression during the early postoperative period, which completely disappeared in three months.

**Figure 3:**
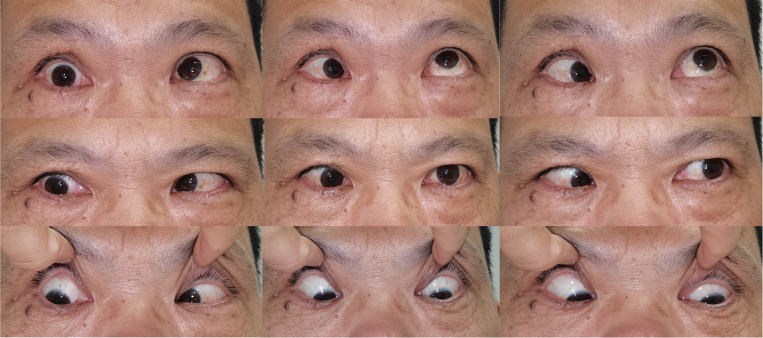
Clinical photograph showing Patient 3 with restricted esotropia and katotropia at a 15-month follow-up.

Patient 3 complained of reduced vision in his right eye. The vision was 0.15OD and 0.8OS with no motility restriction or proptosis. MR showed a mass inferior to the optic nerve (Figure 1E). The lesion was exposed via disinsertion of MRM and identified at the inferomedial quadrant of the intraconal space. The diagnosis was consistent with schwannoma. The vision increased to 0.5OD and 0.8OS two months after complete resection of the tumor. Restriction of adduction and suppression occurred during the immediate postoperative period. Restricted strabismus, however, was detected at a 15-month follow-up (Figure 3).

Patient 4 presented reduced vision and ophthalmalgia in her right eye. The vision was 0.6 OD and 1.2 OS with a slight restriction of adduction and suppression OD. MRI showed the optic nerve was pushed superomedially by a mass with a clear boundary and obvious contrast enhancement (Figures 2A–C). The lesion was accessed between the MRM and IRM in the surgery (Figure 2F). Complete resection was achieved and histopathologic diagnosis was consistent with schwannoma. The patient also experienced transient palsy of MRM and IRM that resolved in 2 months. Visual impairment completely recovered.

Patient 5 was hospitalized with the complaint of insufferable ophthalmalgia in her left eye for 6 months. She had a history of pituitary tumor, for which she underwent microsurgery 11 years ago. Loss of vision in her left eye followed the operation. Physical examination showed loss of pupillary light reflex OS. The vision was 0.6 OD and NPS OS with restriction of elevation, depression, and adduction OS and 3 mm of left proptosis. Imaging showed the lesion involved the left sellar region, suprasellar region, cavernous sinus, intraconal space, infratemporal fossa, and pterygopalatine fossa (Figures 1G,H). Suspicion of the recurrent pituitary tumor was raised. Fully informed of the limited value of surgery in tumor management, the patient insisted on taking a palliative operation in the orbit, and only expected to relieve pain. Endonasal surgery was performed to partially resect the tumor in the intraconal space. Histopathology was consistent with a pituitary endocrine tumor. Remission of ophthalmalgia was achieved postoperatively, after which she turned to Gamma Knife for further treatment.

No other long-term complications have been noted in all five patients with a mean follow-up of 22 (range, 14–32) months. No patients with primary tumors have required additional surgery for tumor recurrence.

### Surgical techniques

Endonasal surgery for all patients was carried out under general anesthesia. All the procedures started with a complete ipsilateral maxillary antrostomy, total ethmoidectomy, and sphenoidotomy to skeletonize the lamina papyracea. Septoplasty was performed according to the necessity. The use of a drill was be utilized to thin the thick orbital floor prior to or instead of fracturing it to expose the underlying periorbita. The lamina was resected and periorbita removed (Figure 2D), after which the orbital fat is carefully removed using low-temperature plasma to identify the extraocular muscles (Figure 2E). The creation of an operative window between the MRM and IRM represents the ideal surgical corridor. The MRM was retracted using three different techniques: (1) an infant eyelid speculum placed between the MRM and IRM, (2) external MRM disinsertion via a transconjunctival incision, and (3) a four-handed method performed transseptally by two surgeons.

**Figure 4:**
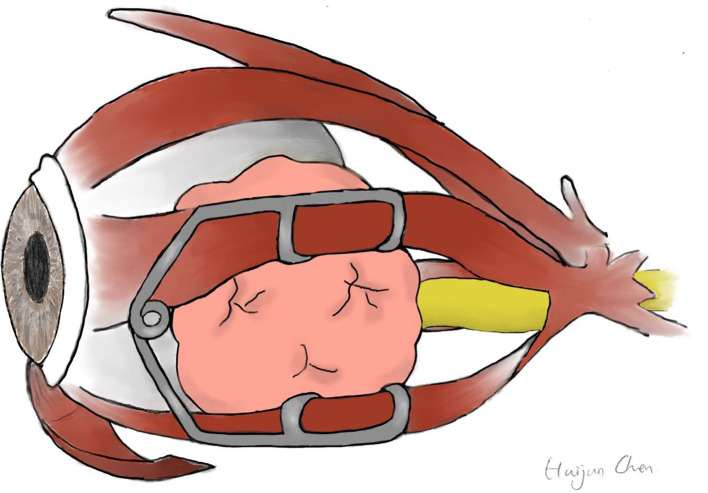
Schematic illustration of an eyelid speculum keeping the MRM and IRM apart.

A wire eyelid speculum worked as an extraocular muscle speculum to keep the MRM and IRM apart (Figures 2F,I). As illustrated in Figure 4, the upper and lower wire blade was placed respectively on the MRM and IRM. The tumor was then approached and resected through this surgical corridor.

**Figure 5:**
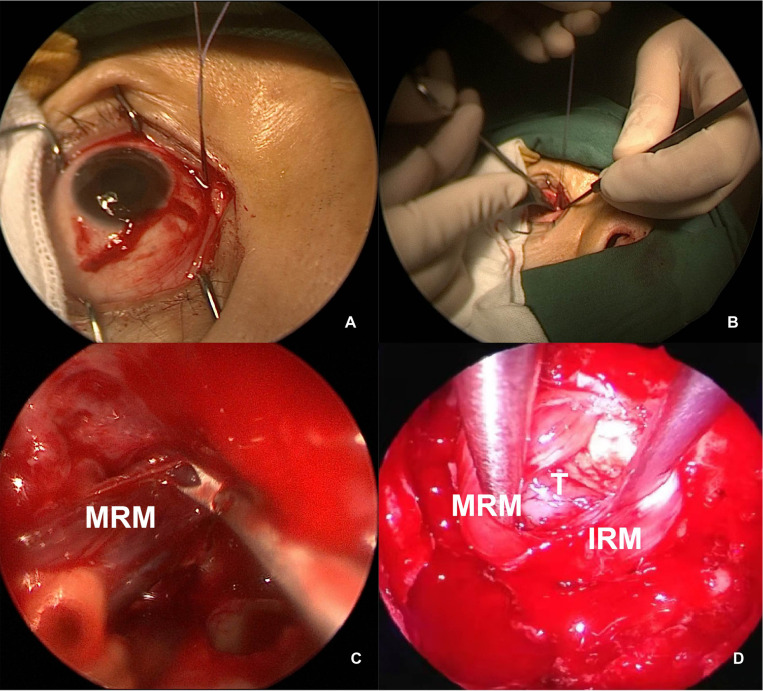
Intraoperative view of MRM retraction. (**A,B**) External view of the right MRM identified and detached, with a stitch looped for control. (**C**) Endoscopic view of the MRM pushed toward the sphenoid sinus. (**D**) The MRM and IRM were retracted transeptally by a second surgeon. MRM, medial rectus muscle; IRM, inferior rectus muscle; T, tumor.

When performing the external technique, we worked in close collaboration with ophthalmic surgeons. An incision was made on the bulbar conjunctiva, and the MRM was detached externally and retracted under the endoscope toward the sphenoid sinus, after being tied by a stitch loop 5 mm away from its ocular insertion on the globe. The reserved stitch loop was placed over the globe for muscle reattachment on completion of tumor resection (Figures 5A–C).

In a four-handed transseptal technique, mid-septal staggered bilateral mucosal incisions were made to allow bilateral access for instruments. One surgeon operated the endoscope and performed dissection in the ipsilateral side, and the other surgeon provided continual suction and retraction with freer elevators in the contralateral side (Figure 5D).

Dissection was performed once the capsule of the tumor was reached, mostly using cottonoids and blunt dissection. The meningioma of Patient 2 in the central orbit encased the intracanalicular segment of the optic nerve with strong adherence. With no hope for vision improvement, the optic nerve was cut off at the orifice of the optical canal to allow for complete resection of the tumor. The schwannoma of Patient 4 originated in the lateral intraconal space and pushed the optic nerve superomedially, thus presenting itself between the MRM and IRM (Figures 2A–C,F). Therefore, the tumor was accessible once the medial intraconal space was exposed by the eyelid speculum. Afterward, complete tumor resection was performed with blunt dissection through this surgical window (Figure 2G).

Low-temperature plasma was cautiously used for hemostasis around the orbit. The medial orbital wall might be simultaneously reconstructed with septal cartilage or a spit middle turbinate graft to prevent diplopia and enophthalmos.

## Discussion

Management of intraorbital tumors involve multidisciplinary expertise. Classically, neurosurgeons and orbital surgeons adopted external approaches including lateral and medial orbitotomies and the transcranial approach. These invasive approaches are associated with serious complications such as cerebral edema, stroke, seizures, CSF leak, and visual compromise (4). More recently, orbital surgeons utilized transconjunctival and transcaruncular approaches which are less invasive with minimal cosmetic disruption (5, 6). All external approaches, however, are significantly limited for accessing intraconal tumors on account that they afford poor visibility and require significant retraction of orbital contents.

The endonasal approach, minimizing external scarring, and enhancing illumination, is now gaining popularity over traditional approaches. Despite a remarkable number of articles focusing on endonasal orbital surgery, the literature on this topic is still quite heterogeneous. In general, a consensus has been reached lesions medial and/or inferior to the optic nerve can be accessed safely endoscopically. As surgeons become more comfortable with the endoscopic approach, the indications may expand to include lesions lateral to the optic nerve. The cavernous hemangioma exclusively endonasal resection (CHEER) staging system defined the limits of an EER and established five stages based on anatomic relationships of the tumor to critical orbital structures and foramina (7). The system stated that tumors considered amenable for an exclusively endonasal resection should be located medial to a plane along the long axis of the optic nerve, or in select cases, extending inferolaterally but remaining below a plane from the contralateral naris through the long axis of the optic nerve. In our study, Patient 4 with a tumor lateral to the optic nerve had increased visual acuity without any long-term complications postoperatively. The optic nerve of Patient 4, however, was superomedially located due to tumor volumetric increase (Figures 4A–C), for which the stage of the tumor could not be exactly categorized by the CHEER staging system. The real challenge is when the optic nerve is in its natural position and the tumor is located lateral to it. At the time of writing, no gold standard has been established for intraorbital tumors lateral to the optic nerve. Dziedzic et al. described a successful case of the endoscopic endonasal transmaxillary, transpterygoidal approach to remove an extraconal tumor located lateral to the optic nerve in the orbital apex (8). Dallan et al. also presented promising results of an endoscopic-assisted superior-eyelid approach for the management of selected supero-lateral intraorbital lesions (9). With only one successful case of the endonasally-treated lateral intraconal lesion, we do not want to revolutionize the indication of endonasal orbital surgery, but we would simply describe our ongoing experience, adding our cases to the current body of data on this topic.

Within the orbit, the intraconal space is especially challenging to address due to the complexity of the anatomy and varying relationship of the tumor to critical neurovascular structures and skull-base foramina (10). A thorough understanding of the intraconal anatomy is paramount to help minimize the risk of injury to the neurovascular structures. A high-resolution computed tomography scan accesses the paranasal sinuses and bony structures. Enhanced MRI helps the surgeon assess the character of the lesion as well as the site of the optic nerve and ophthalmic artery, which can be highly variable (11, 12). With these techniques laying the foundation, the use of fused image navigation improves accuracy when pinpointing lesions and minimizes injury to crucial structures intraoperatively (13).

Apart from the complex anatomy of intraconal space, Extraocular muscles, especially the MRM, set up the barrier against the nasal cavity, further increasing the difficulty of exposure of and manipulation in this area. Multiple methods of MRM retraction have been used based on individual surgeon experience and preference. McKinney et al. (14) described external MRM retraction at the globe insertion point by using a vessel loop. Retraction of the MRM toward the choana with 2–0 silk thread has been described by a number of researchers that noted favorable exposure (15). Using this technique, applying just enough gentle traction allows for exposure of the orbital apex. A similar technique of placing a vessel loop with a suture instrument around the MRM through a controlled septotomy has also been described (16, 17). Placement of circumferential external or internal, however, exerts tension on innervation and blood supply to MRM which was adjacent to its annular insertion. A four-handed technique with endoscopic instrumentation reduces the tension by allowing the muscle to relax during endoscope and instrument changes (18–20). Lin et al. (21) evaluated each of the reported methods of MRM retraction on eight orbits from four cadaver heads and quantified the degree of intraconal exposure. The conclusion was drawn that both transseptal MRM retraction was favorable and provided the largest total area of exposure to medial intraconal space.

Herein, we adopted three different techniques of MRM retraction without any visual impairment or significant morbidity. Use of the four-handed approach allowed for dynamic adjustments in retraction and protected the neurovascular inputs of the MRM. While this technique avoids damages to the MRM, the optimal method of the retraction remains elusive and necessitates expert cooperation. Disinsertion of the MRM better facilitated exposure because the boundary of intraconal space was temporarily removed at the cost of a conjunctival incision and a worse outcome, usually applying an ophthalmologic surgeon. Eyelid specula are originally used to keep the lids apart in intraocular or extraocular surgery. Our study utilized an eyelid speculum to retract extraocular muscles, and its application value was surprisingly confirmed. Use of an ocular speculum may set free a second operating hand either holding an endoscopic instrument or pulling the end of silk threads, thus expanding the intranasal space of operation. The four-handed approach and the ocular speculum method only resulted in transient ophthalmoplegia, providing a less eventful postoperative course compared to external disinsertion of the MRM. The comparison of three exposing methods was summarized in Table 2.

**Table 2:** Comparison of MRM retraction techniques.

	Indication	Advantage	Disadvantage
Four-handed way	Intraconal lesions	Less tension on MRM, dynamic manipulation	Requirement of cooperation
Disinsertion		Best exposure	Ocular motility restriction, a conjunctival incision
Speculum		Less tension on MRM, expanding space of operation	Not special for extraocular muscles

*MRM, medial rectus muscle.*

Our preliminary results of the MRM retraction method with an eyelid speculum are promising with successful functional and cosmetic outcomes and reduced morbidity for the patient. This technique should be considered as an option for selected intraconal lesions. An extraocular muscle speculum can be specially made that comes in different sizes to better suit MRM retraction. We are well aware that larger case series are needed in an effort to understand the real applicability of such a technique.

## Conclusion

Endoscopic techniques provide superior access to intraconal lesions. This approach is safe, effective, and offers marked advantages over traditional techniques. The field is still expanding and the surgical indication of endoscopic resection will continue to be challenged. An eyelid speculum enhances surgical access, and eases the pressure exerted on extraocular muscles, which has a promising application prospect.

## Data Availability

The datasets presented in this study can be found in online repositories. The names of the repository/repositories and accession number(s) can be found in the article/Supplementary Material.
